# Potential clinical treatment prospects behind the molecular mechanism of alternative lengthening of telomeres (ALT)

**DOI:** 10.7150/jca.80097

**Published:** 2023-01-31

**Authors:** Haolu Sun, Guijuan Chen, Baochang Guo, Shushu Lv, Guojun Yuan

**Affiliations:** 1 School of Environment and Chemical Engineering, Anhui Vocational and Technical College, Hefei, 230011, China.; 2 Department of Pathophysiology, School of Basic Medical Sciences, Anhui Medical University, Hefei, 230011, China.; 3 Rehabilitation Department of Traditional Chinese Medicine, 969 Hospital of the Joint Support Force of the Chinese People's Liberation Army, Hohhot, 010000, China.; 4 Department of Pathology, The First Affiliated Hospital of Huzhou University, Huzhou 313000, China.

**Keywords:** Telomerase, ALT, molecular mechanism, diseases, therapeutic targets

## Abstract

Normal somatic cells inevitably experience replicative stress and senescence during proliferation. Somatic cell carcinogenesis can be prevented in part by limiting the reproduction of damaged or old cells and removing them from the cell cycle [1, 2]. However, Cancer cells must overcome the issues of replication pressure and senescence as well as preserve telomere length in order to achieve immortality, in contrast to normal somatic cells [1, 2]. Although telomerase accounts for the bulk of telomere lengthening methods in human cancer cells, there is a non-negligible portion of telomere lengthening pathways that depend on alternative lengthening of telomeres (ALT) [3]. For the selection of novel possible therapeutic targets for ALT-related disorders, a thorough understanding of the molecular biology of these diseases is crucial [4]. The roles of ALT, typical ALT tumor cell traits, the pathophysiology and molecular mechanisms of ALT tumor disorders, such as adrenocortical carcinoma (ACC), are all summarized in this work. Additionally, this research compiles as many of its hypothetically viable but unproven treatment targets as it can (ALT-associated PML bodies (APB), etc.). This review is intended to contribute as much as possible to the development of research, while also trying to provide a partial information for prospective investigations on ALT pathways and associated diseases.

## Introduction

Telomeres are short sections of DNA-protein complexes found at the ends of linear chromosomes in eukaryotic cells. Telomeres are shielded by complex proteins, which prevents them from being identified as DNA double-strand breaks (DSBs) 4. Telomeres shorten with each cell cycle as a result of lagging replication fork strands' inability to duplicate telomere ends during somatic cell division 5. We can anticipate that telomere attrition will eventually result in cellular senescence, which is a barrier to carcinogenesis, if cells continue to proliferate 1, 2. Cancer is characterized by genomic instability, and DNA replication is the biological mechanism most likely to cause instability. Replication stress, a cause of genomic instability and a trait of precancerous and cancerous cells, is caused by any circumstance that produces a lot of DNA damage 6. Under normal circumstances, the body restricts replication stress cells reproduction and halts the cell cycle 6. Therefore, to obtain eternal life by preserving telomere length during proliferation, cancer cells must overcome replication stress and senescence 2, 5. A small number of human cancers, such as adrenocortical carcinoma, neuroblastoma cell tumor, osteosarcoma, and astrocytoma, rely on the alternative lengthening of telomerase (ALT) pathway to cause telomere shortening, despite the fact that the majority of human malignancies (>85%) use telomerase to lengthen telomeres 3, 7-9.

Some cancer types have been reported to contain ALT+, and ALT may offer new possibilities for the next clinical cancer studies. The development of new clinical medicines is based on molecular biological research on ALT tumors, which is also required for the diagnosis and management of ALT tumors and other related disorders. This article seeks to provide information for researchers to investigate ALT tumors and associated diseases by summarizing the primary mechanisms of telomere lengthening and ALT tumor diseases as they are now understood.

## What is the ALT mechanism?

Many of the most resistant cancer subtypes exhibit ALT, a telomere preservation mechanism 10. As we discuss ALT, we have to introduce telomeres. Telomeres become important in preserving the stability of the human DNA 3. First of all, telomeres shield linear chromosomal ends from being identified as DNADSBs 11. Then, through the start of cellular senescence, the increasing wear of telomeres that results from each round of cell division has the power to suppress malignancies and restrict cell division 3, 12. Activating the telomere maintenance mechanism (TMM) allows tumor cells to multiply quickly and become immortal 3. To achieve TMM, 85-90% of these tumor cells activate telomerase 13. However, the others 10%-15% of tumor cells through the ALT mechanism 3.

## Telomeres structures and ALT tumor cells' typical property

### Telomeres structures

Telomeres are conserved nucleoprotein structures localized at the ends of eukaryotic linear chromosomes. (1) Double-stranded TTAGGG repeats can be found in the genetic material 14. (2) Telomere-associated proteins, such as the Shelterin complex 14. Telomere repeat binding factor 1 (TRF1), telomere repeat binding factor 2 (TRF2), TRF1-interacting nuclear protein 1 (TIN1), repressor-activator protein 1 homolog (RAP1), tripeptidyl-peptididase 1 (TPP1), and protection of telomere 1 (POT1) are the components of shelterin 15.

### ALT cells exhibit a variety of typical traits

**Figure 1:**
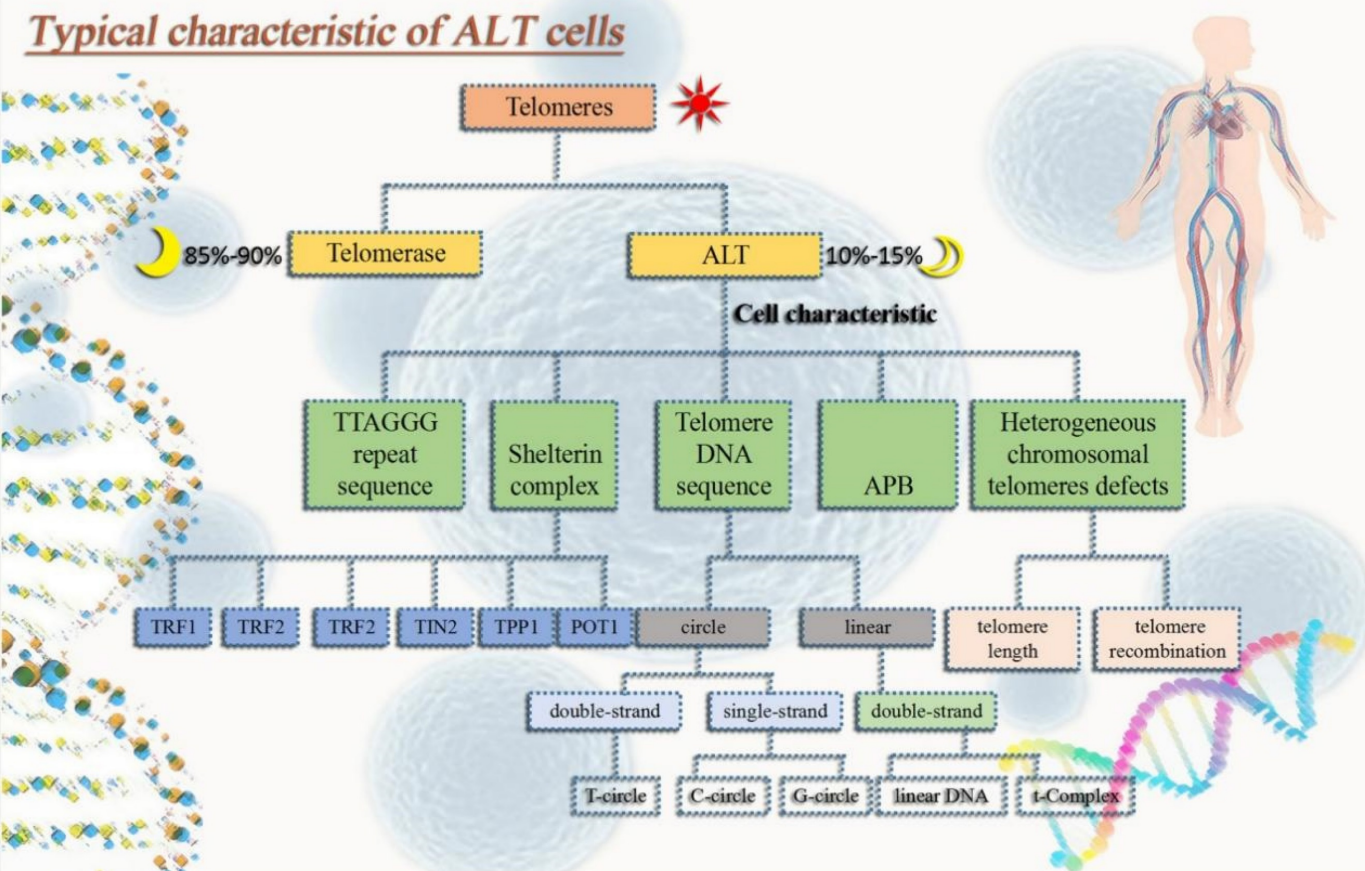
Typical characteristic of ALT tumor cells. In order to avoid aging and gain immortality, tumor cells usually extend telomeres in two pathways, one is telomerase and the other one is ALT. ALT tumor cells have typical properties that not only help us identify whether ALT mechanisms occur in tumor cells, but also in some ways help maintain ALT tumor cell homeostasis.

(1) Extrachromosomal telomeric repeats DNA (ECTRs). Mostly telomere circle (t-circle) are included 16, single-stranded circles in part (called C-circle or G-circle depending on whether it is rich in C or G) 17, 18, T-Complex DNA with a very high molecular weight and linear double-stranded DNA 18. (2) One distinguishing characteristic of ALT cells is the promyelocytic leukemia nucleosome (PML nucleosome), which contains telomere chromatin. It is known as the ALT-related PML body (APB) as a result 19. (3) Heterogeneous telomere length. Chromosomal telomeres with a high degree of heterogeneity can lead to stability problems. Highly heterogeneous chromosomal telomeres provide for rapid changes in telomere length 20 and a dramatic increase in telomere recombination rates 19, 21. (4) High level of telomere-sister chromatid exchanges (T-SCEs) 22** (Fig. 1).**

### Markers and methods for the identification of ALT

**Figure 2:**
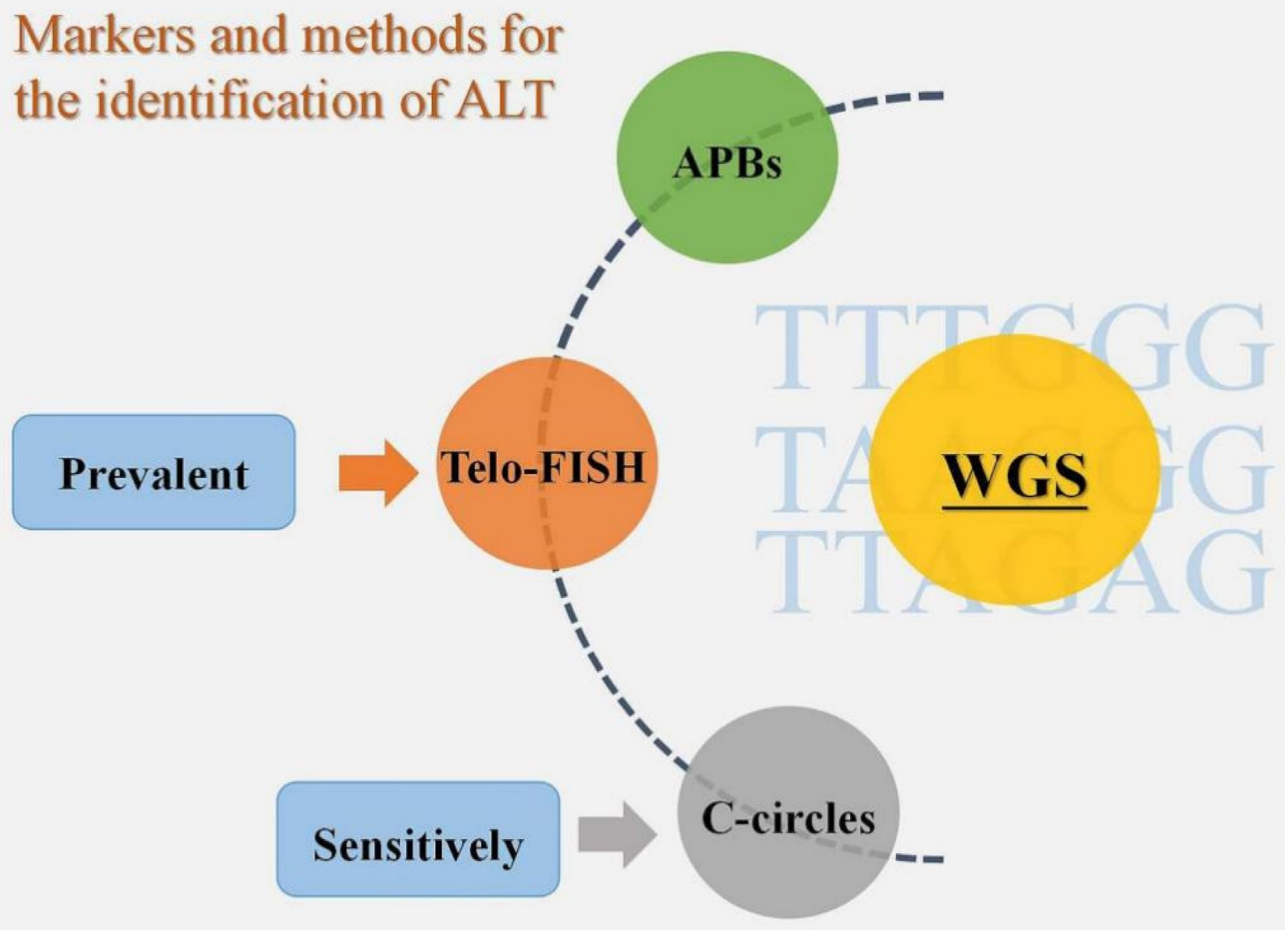
Markers and methods for the identification of ALT. APBs, Telo-FISH, and C-circles are the three biomarkers that have so far been used to diagnose ALT+ tumors, with C-circles being the most sensitive and Telo-FISH being the most popular. WGS, however, is a new technology that may offer greater convenience and accuracy.

According to the review of earlier studies, there is currently no unambiguous agreement on ALT identification tactics. APBs, Telo-FISH, and C-circles have all been employed extensively in the past as biomarkers for ALT detection 23. There is proof that the most sensitive ALT biomarker among them is C-circles 17. Additionally, Telo-FISH was the most often published technique for ALT detection in cohort studies using sizable tumor sample sets 23. In reality, to guarantee the identification of ALT, we need to apply two or more biomarker identification techniques, regardless of the method we select - Telo-FISH, C-circles, or APBs 23. A solid option for TMM identification technique is also accessible, based on the varied phenotype of ALT, and it may entail the use of a number of biomarkers, such as CCA, APB, and TIF. Compared to conventional identification methods, the introduction of whole genome sequencing (WGS) has led to new concepts for the identification of TMM 24. According to one study, WGS may accurately predict tumor TMM by validating the frequency of telomere variant repeats, particularly TTTGGG, TAAGGG, and TTAGAG sequences 24. The accuracy of WGS in identifying tumor TMM was determined to be 91.6% based on the validation data 24. The variable repeat content was also found to be sufficient to identify whether a tumor was ALT-positive or ALT-negative based on sequencing a sizable sample of 21 tumor subtypes (n = 821 samples), and this technique may develop into a novel and promising one for ALT+ tumor detection 23** (Fig. 2).**

### Molecular mechanism of ALT cells

On one hand, current studies have shown that at least two types of ALT mechanisms exist. the RAD51-dependent type I and the RAD59-dependent (or RAD51-independent) type II 25. However, RAD59 is not present in mammals 26. However, RAD59 is involved in the RAD51- and RAD54-independent break-induced replication (BIR) pathways, suggesting that type II telomerase deficient yeast survivors may use the RAD51-independent BIR pathway for telomere lengthening 27. It is now widely believed that the RAD51-independent BIR pathway may be more central in mediating ALT 25. Similar to the yeast type I ALT system, the homologous recombination-dependent ALT pathway in human cancer is a RAD51-mediated mechanism that needs the MRE11-RAD50-NBS1 (MRN) recombination complex to be intact 28-30. On the other hand, within the nucleus of eukaryotes, the genome is a dynamic, non-randomly ordered structure. Organized into active/inactive regions, membrane-free bodies, thin-layered associated domains, protein- or RNA-mediated loops, enhancer-promoter contacts, and chromatin regions with varying accessibility, chromatin is organized into a complex, highly hierarchical three-dimensional structural network 31-33. To maintain and sustain the viability of cellular processes, epigenetic and transcriptional mechanisms that are carefully regulated in location and time create this complex chromatin structure 34. The chromatin structure also divides the genome into constitutive heterochromatin regions, which are characterized by cohesive chromatin fibers, high levels of DNA and histone methylation, and transcriptional repression of the underlying DNA sequence 34. These regions are large repetitive and gene-poor regions 34. Telomeres are repeated structures found at the ends of chromosomes that are epistemically kept in a repressed heterochromatic state. This prevents DSBs from being recognized, prevents DNA damage repair, and promotes cell growth 35-37. The production of Telomere-repeat-containing RNA (TERRA) and a low density of histone methylation are two characteristics of the non-classical, relaxed epigenetic state that telomeres adopt in some cancer cells 35-37. TERRA is connected to telomere stability, telomere heterochromatin development, and telomerase regulation 38, 39. According to earlier research, TERRA attaches to extra telomere chromatin and influences the transcription of neighboring genes. TERRA is also associated with a proteome that is engaged in a number of processes, including chromatin remodeling and transcription 40, 41. Telomeres in yeast and human cells are vulnerable to DSB and homology-directed repair (HDR) because of the TERRA R-loop established there 36, 37, 42. Without telomerase, HDR may in some circumstances be able to cause telomere lengthening 43, 44. As a result, it is now thought that TERRA could cause ALT to initiate.

**Figure 3:**
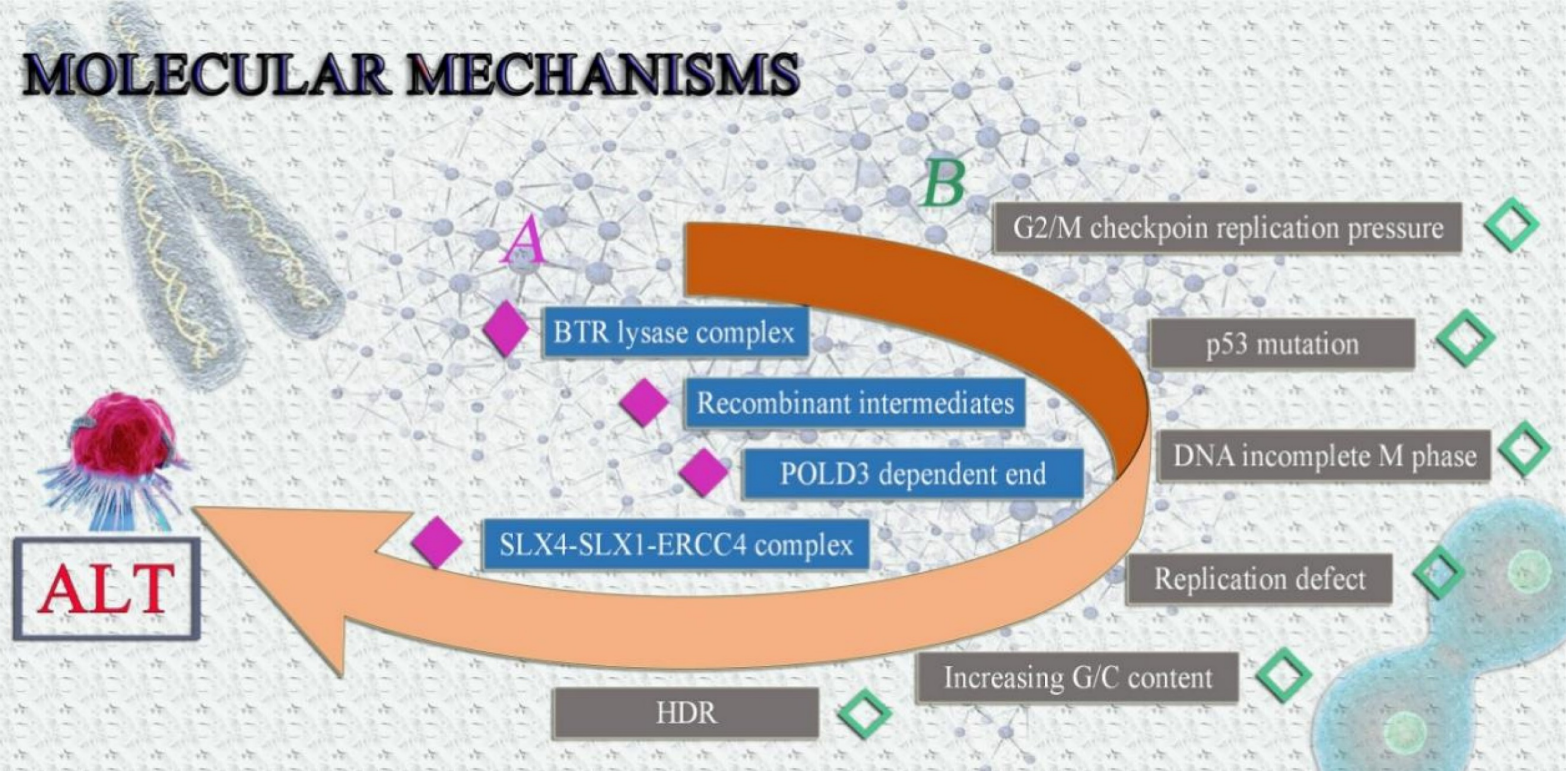
Possible molecular mechanisms of ALT tumor cells. It is currently believed that the formation of ALT involves two possible molecular mechanisms, both of which can lead to the extension of telomeres in tumor cells in the absence of telomerase, avoiding senescence and prolonging survival.

The following are some potential ALT mechanisms: (1) The BLM-TOP3A-RMI(BTR) lysase complex is necessary for ALT-mediated telomere extent 28. During the chain invasion, the creation of recombinant intermediates is suggested 28. Similar to this, chain invasion causes the fast and expansive POLD3-dependent end to form 45. Finally, the SLX4-SLX1-ERCC4 complex uses granular synthesis to speed up resolving recombination intermediates, leading to telomere exchange without telomere extension 46. (2) Telomere mitotic DNA synthesis (MiDAS) is a conservative DNA synthesis method that builds up replication pressure at G2/M checkpoints in malfunctioning cells 47. The majority of ALT tumor cells frequently harbor *p53* and G2/M checkpoint mutations 48, 49. Checkpoint and *p53* mutation may be the primary causes of the genomic instability of ALT tumor cells, according to conjecture 49, 50. Because the ALT tumor cells' genomes are unstable, cellular DNA is easily triggered to enter the incomplete M phase, build up synthetic replication pressure, and result in more replication defects 28, 51. Increased G/C content is found in telomere sequences and secondary structures, such as the creation of G-quadruplexes and R-loops, which induce HDR and set off ALT, as a result of replication errors and persistent DNA damage responses 28** (Fig. 3).**

## The relationship between the ALT mechanism and medical conditions

### ALT incidence in various cancer types

Even though 80-85% of TMM positive cancers show telomerase activity, but, according to many excellent earlier studies, tumors originating in mesenchymal tissues like bone, soft tissue, the neuroendocrine system, the peripheral nervous system, and the central nervous system are typically marked by ALT activity 52. According to certain data, the prevalence of osteosarcoma ALT+ might reach 64% and 9% in synovial sarcoma 53, 54. Additionally, ALT+ rates in soft tissue tumors were higher than we had anticipated, at 62%, 58%, and 25% for malignant fibrous histiocytoma, leiomyosarcoma, and liposarcoma, respectively 53-56. However, more than 50% of tumor cells in the neuroendocrine system employed ALT to prolong telomeres. PanNET made up 53% of them, Paraganglioma 13%, and Carcinoid tumor 6% 53, 55, 57, 58. The central nervous system and the peripheral nervous system should both be given substantial consideration because they are the "hardest hit" by ALT+ 8, 53, 55. With 34% of ALT+ in the peripheral nerve system, neuroblastoma takes the top spot, followed by ganglioneuroblastoma (14%), and adrenocortical carcinoma (12%) 8, 53, 55. Astrocytoma and glioblastoma both exhibit high ALT+ rates in the central nervous system—42% and 28%, respectively 53, 55, 59. The ALT+ in the gastrointestinal system is also of value, with MSI-H Gastric Carcinoma and Non-MSI-H Gastric Carcinoma having 57% and 19% positive rates, respectively, and Gastric Carcinoma having a 19% ALT+ rate 53, 60. Additionally, 6% of intestinal cancers exhibited ALT+ 57. We discovered that ALT has been detected in many different tumor forms and has been positive in more than half of some cancers based on the investigation of ALT incidence in different cancer types. Therefore, it is key for clinical practice to investigate ALT's mechanism.

### Non-small cell lung cancer (NSCLC)

With an estimated 22 million new cases and 17.9 million deaths per year, lung cancer is one of the most common malignancies and the main cause of cancer-related deaths globally 61. There have been considerable advancements and gains in survival for many patients as a result of the present research of disease biology, the use of prognostic biomarkers, and improvements in treatment 61. However, public health initiatives to lower smoking rates have dramatically decreased lung cancer incidence in developed nations 62. Although numerous studies have looked at the disease process in NSCLC, one of the most prevalent types of lung cancer, there are few studies looking into the mechanisms of ALT 63. Despite this, the number of new lung cancer diagnoses rises every year in low-income nations 63-65. In one study, 16 human NSCLC cell lines were used to examine the mechanism of telomere stabilization 66. The results revealed that the majority of the cancer cell lines were TA-positive 66. The TA-negative NSCLC cell line SK-LU-1, on the other hand, exhibited all ALT characteristics, including APB and typical TRF length heterogeneity 66. Surprisingly, we were unable to identify ALT characteristics in two other NSCLC cell lines, SK-LU-1 and *hTERT* mRNA-negative TA and *hTERT* 67, 68. It is common knowledge that the ALT machinery is believed to involve recombination processes that result in heterogeneous and extra-long telomeres that are comparable to those reported in yeast telomerase negative type II survivors 69, 70. Additionally, it was discovered that TA deficiency was not related with decreased proliferation and tumorigenicity if the cells displayed ALT features during *in vitro* tests of NSCLC cell lines 66. These findings imply that telomerase directly contributes to the NSCLC cells' rapid development 66. The researchers also discovered that SCID mice and TA-negative NSCLC cell lines [3 of 16 (19%)] showed significantly reduced invasive growth 66. *In vitro* and animal evidence, which all demonstrated a noticeably worse prognosis in lung cancer patients expressing TA and/or *hTERT*, particularly in stage I NSCLC, supported the conclusions of multiple clinical trials 71-74. Furthermore, TA and/or *hTERT* expression were linked to improved clinical staging, according to clinical investigations 75. A much worse prognosis based on a combination of recombination events involving extremely long telomeres and enhanced genomic instability based on recombination events involving very short telomeres was blamed for the good prognosis of patients with ALT-positive malignancies 76.

### Breast cancer

Breast cancer accounts for 18% of all female cancer cases and is the most frequent malignancy in women worldwide 77. The most frequent histologic type of breast cancer, accounting for about 68% of cases, is invasive ductal carcinoma 78. Four primary breast cancer subgroups have been identified as a result of a new classification of breast cancers based on DNA microarray gene expression profiles 79. These subgroups appear to originate from different cell types with various biological activities. These include subtype A (defined as significant ER and ductal epithelium-related gene expression and low histologic grade), subtype B (defined as lower ER expression and higher histologic grade), subtype C HER-2 positive (defined as low ER and HER-2 expression and high HER-2 expression), and subtype D basal-like (defined as low ER and HER-2 expression and high myoepithelial expression) 79. Transmembrane tyrosine kinase growth factor receptor ERBB2 is a proto-oncogene that is located on chromosome 17q and that, in 15-20% of invasive breast tumors, amplifies, leading to overexpression of the HER-2 receptor 80. The tumor suppressor genes *p53* and *BRCA1* as well as the topoisomerase IIα gene are situated on chromosome 17, along with a number of other genes involved in the development of breast cancer 81. The ALT phenotype is uncommon in breast cancer but nearly never seen in other malignancies, according to this tiny study 82. However, it does appear preferentially in a subset of HER-2 overexpressed breast tumors 81. High levels of gene amplification are a well-known feature of HER-2 positive breast tumors, which may indicate a higher level of genomic complexity and change 81. The replication and recombination events caused by breaks appear to be how the ALT mechanism works 82, 83. These activities could result in free chromosome ends that engage in end-to-end association and trigger break-fusion bridge cycles, which would increase the frequency of complicated chromosomal rearrangements 82, 83. A link between SLX4-interacting protein (SLX4IP) and TMMs has also been identified 83. It has been demonstrated that SLX4IP is a key regulator of metastatic recurrence in breast cancer metastasis and recurrence. When SLX4IP is inactive, ALT is inhibited, and telomerase is activated simultaneously 83. In individuals with genetically different breast cancer subtypes, TMMs selection has a significant impact on metastatic progression and survival 83, 84. In particular, TMM was pharmacologically and genetically modulated in disseminated tumor cells, causing telomere-dependent cell death and preventing disease recurrence 84.

### Prostate cancer

With more than 160,000 cases and 26,000 fatalities per year in the United States, prostate cancer is the second most frequent cancer in males 85. Prostate cancer cells, like those of other cancers, must avoid replicative senescence and the potentially fatal chromosomal instability brought on by telomere malfunction 69. Recurrent cancer-specific somatic inactivating mutations in the *ATRX-DAXX* chromatin remodeling complex are strongly correlated with the presence of ALT 86, 87. It has been demonstrated that deletion of *ATRX* is sufficient to cause several ALT-related characteristics, including the existence of ALT, in LAPC-4 cell lines 88. *ATRX* deletion is crucial for ALT activation, as shown by the development of genuine ALT in adenocarcinoma cells after *ATRX* inactivation and subsequent loss of telomerase activity 88. That is, the inactivation of important ALT suppressors like *ATRX* can change telomere maintenance from telomerase to ALT under the selection pressure of loss of telomerase activity and in the appropriate genetic and/or epigenetic background 89. Additionally, it has been documented in the literature that TRF2 is crucial to the pathological development of prostate tumors and that prostate tumors with low levels of TRF2 expression are categorized as high-grade androgen receptor-negative tumors 85, 90. High tumorigenicity and telomere maintenance are provided by prostate cancer stem cells with diminished TRF2 expression via telomerase and ALT 85. However, the study also found that prostate cancers that survived in the presence of TRF2 and Terc deficiency lacked both telomerase activity and the ALT marker APB 85. As a result, the researchers hypothesize that prostate cancers may have telomere maintenance mechanisms other than telomerase and ALT processes 85.

### Adrenocortical cancer (ACC)

Telomerase activity (TA) and alpha-thymidine kinase are two distinct TMM processes in ACC, according to the high-quality ACC tissue assessment findings gathered by the University of Michigan Health System 8. Alternative telomere lengthening methods were assessed by telomere restriction fragment analysis (TRF) 91. To some extent, telomere length is a representation of TA 8. The "gold standard" for measuring telomere length is TRF analysis, which was created based on the understanding that TTAGGG sequences are highly conserved 92-94. By assessing the intensity of telomere smears, TRF analysis calculates the average telomere length 92-94. The repeating telomere sequences (TTAGGG), which do not contain the sites of the restriction enzymes utilized, do not break down when genomic DNA is digested 91. Consequently, depending on where the restriction enzyme digested submonomeric region is located, each TRF will have a specific number of non-monomeric sequences (referred to as X-regions) 93. Following DNA digestion, gel electrophoresis is carried out, and Southern blot analysis can be used to identify telomere sequences 93. The findings demonstrated that ACC involves activation of the ALT mechanism 8.

### Neuroblastoma

An embryonal tumor of the autonomic nervous system called neuroblastoma. The neural crest tissue's growing precursor cells and partially committed precursor cells are assumed to be the source of primordial cells 95. High polymer content, low telomerase reverse transcriptase (*TERT*) expression, and enhanced TERRA expression are all hallmarks of ALT cells 96, 97. One of the ALT tumors is the neuroblastoma. More than 50% of ALT-positive neuroblastoma cells had an *ATRX*-linked mutation, which is associated with -thalassemia. The telomeres of ALT-positive malignancies are also enriched with the heterochromatin histone marker H3K9me3 that *ATRX* recognizes 7. It is generally accepted that *ATRX* mutations or lower expression levels of *ATRX*/death domain-associated proteins (*ATRX*/*DAXX*) in cells are responsible for the phenotypic shift in ALT cells 7. The reduction of *ATRX* level in ALT *ATRX* wild-type neuroblastoma can be accomplished by lowering *DAXX* level 7. Reduced *DAXX* levels cause orphan *ATRX* protein molecules to degrade and prevent the formation of the *ATRX*/*DAXX* complex 7. The ALT phenotype in neuroblastoma has been linked to *ATRX* genome changes in several studies, however the linkage between ALT and *ATRX* genome changes has not been clearly shown (using particular markers) 98. 60% of ALT cancers had *ATRX* structural variations, framework fusions, and single nucleotide mutations 98. Regardless of their *ATRX* status, patients with the ALT phenotype have poor overall survival in practice, despite these associations 99, 100.

Truly high-risk tumors are those that are TMM-positive, *TERT*-high-ALT, or both 98. According to the findings 100, *TERT* activation in high-risk neuroblastoma is not just present in *TERT*SV+ and *MYCN*-amplified tumors. Interestingly, some tumor cells that did not undergo the aforementioned transformation also expressed *TERT* highly 100. APB, C-circle, and *TERT* expression levels were measured by a series of studies. The findings revealed that 12% to 26% of high-risk neuroblastoma tumors, including several *MYCN*-Amp tumors, had low *TERT* expression and no ALT activation 101. The patient's overall survival is also considerably higher than that of high-*TERT* patients 98.

### Osteosarcoma

The most prevalent primary solid malignant bone tumor, osteosarcoma generates malignant bone mesenchymal cells and/or immature bone 102, 103. According to statistics, there are between 2 and 3 million new cases of osteosarcoma each year, with youth having the highest incidence 104, 105. The incidence of people aged 15 to 19 could reach 8 to 11 million annually during the peak period 104, 105. According to historical data, men are 1.4 times more likely than women to develop osteosarcoma 103. Approximately 60% of osteosarcoma sample contain the ALT mechanism 106. Recent genomic investigations have found that Osteosarcoma tumors exhibit a high level of structural diversity, including somatic mutations, copy number changes, and different single nucleotide variants or recurring point mutations 107, 108. Recurrent somatic changes are also common in other putative driver genes, such as *ATRX*, while *TP53* and *RB1* genes are the most frequently affected in osteosarcoma 109. *ATRX* was one of the most frequently mutated genes among the 288 osteosarcoma patients examined by the American Association for Cancer Research Genomics Project, second only to *TP53* 110-112. Importantly, because current next-generation sequencing tools cannot detect many complicated variations with short sequence reads, the true incidence of *ATRX* mutations may be underestimated 113. *ATRX* is really among a number of oncogenic driver genes with frequent somatic complex differentials in a variety of cancer types of tumors 113. The relationship between the chromatin remodeling gene *ATRX*, the histone chaperone *DAXX*, and the histone variation H3.3 and the ALT status is well known 101, 114. The promotion of the histone variation H3.3 by *ATRX* and *DAXX* in heterochromatin regions raises the possibility that abnormalities in the stability of telomere heterochromatin can result from the loss of *ATRX*, *DAXX*, and/or H3.3 87, 115, 116. *ATRX*, *DAXX*, and H3.3 gene alterations were discovered in ALT-positive cancer samples, according to prior research, and some of these ALT-positive samples also displayed loss of *ATRX* or *DAXX* expression or localization 101, 114, 115. Here, we identified a new gene fusion event between the kinesin motor protein KIFC3 and *DAXX* using next-generation sequencing, which resulted in the translation of the chimeric *DAXX*-KIFC3 fusion protein 106. The expression of the *DAXX*-KIFC3 fusion protein is activated by the translocation between the untranslated region of the *DAXX* gene and the central intron of KIFC3, a kinesin family member, which results in functional deficiencies in the *DAXX* protein and helps to activate the ALT pathway 106. Other investigations have revealed that the *DAXX* gene locus on chromosome 6p21, the KIFC3 gene locus on chromosome 16q21, and cells production on chromosome 6p21 have all been identified as common fragile locations in the genome 117, 118. This finding suggests that the aforementioned areas are unstable and may play a significant role in the final genomic rearrangement and DSB 117, 118.

We looked for molecular proof of TA and ALT in 62 patient osteosarcoma samples. In this cohort of osteosarcoma patients, Kaplan-Meier analysis revealed that the absence of both TA and ALT (18%) was more strongly related with increased survival (P = 0.05) than staging (P = 0.16) or treatment response (P 0.18). However, Kaplan-Meier analysis also revealed that there was no discernible difference in survival between individuals with ALT+ and ALT- osteosarcoma. The survival rate of patients with ALT+ osteosarcoma was the same as or worse than ALT- group 54, 119, despite the fact that the median follow-up time (28 months) was brief. Analyzing whether the ALT status of the osteosarcoma was connected to treatment response concurrently. Chemotherapy had a 35% ALT+ response rate and a 33% ALT- response rate, but the difference was not statistically significant 54.

### Astrocytoma

The most prevalent tumors of the central nervous system (CNS) are astrocytomas, which are primarily made up of astrocyte-like cells) 120. Astrocytomas are categorized into grades II, III, and IV using the World Health Organization (WHO) system. Glioblastoma (GBM) is another name for grade IV cancer 120, 121. Grade II to IV astrocytomas invade the brain widely and are physiologically malignant 122. The median survival period for GBM is less than one year, making it one of the most aggressive tumors in human cancers 120, 122. Unfortunately, they will eventually experience neurological malfunction and pass away despite advancements in neurosurgery, radiation, and chemotherapy 123, 124.

Clinically, it is common practice to employ *TERT*p and Isocitrate dehydrogenase (*IDH*) mutations to categorize 80% of GBM into genetic subgroups with various clinical trajectories to aid in diagnosis 125-127. The strategies used for telomere maintenance vary according on the subgroup of GBM cellular molecules 54. The development of the initiating transcription factor binding site led to an increase in *TERT* expression, telomerase activation in the *TERT*p mutant GBM, and ALT in the *IDH* mutant GBM as a result of the concurrent loss of *ATRX* function or mutation 55, 128. According to these models, genetic modifications to preserve telomeres may be a crucial stage in the development of glioma cells 9. *ATRX* (particularly without *IDH* or *TP53* mutations) or GBM with mutations in the SWItch/Sucrose Non-Fermentable (SWI/SNF)-related, matrix associated, actin-dependent regulator of chromatin subfamily A-like 1 (*SMARCAL1*) include an ALT-positive *TERT*p^WT^-*IDH*^WT^GBM subgroup known as *IDH*^WT^-ALT 9. An adenosine triphosphate (ATP)-dependent annealing helicase known as *SMARCAL1*, a new potential driver, is involved in catalyzing the recombination of DNA at the junction of the halted replication fork. Replication Protein A (RPA) 129. It is now known to play a role in easing the replication stress caused by telomere recombination. RPA attracts *SMARCAL1* to DNA 9, 130. DNA deterioration and replication at the location where the fork has halted encourage fork repair and restart, preserving the stability of the genome 9. The study's findings suggest that the intact *SMARCAL1* helicase domain is crucial in preventing the production of C-circle and other markers in *SMARCAL1* mutants and cancer cells that are ALT-positive 9. By looking up H3.3^G34R^ and*IDH1*^R132H^ in the public cancer genomes database, other studies have demonstrated 131 that they are thought of as collaborating genes of *ATRX*. Together, they have an impact on the ALT mutations that may occur in glioblastoma patients who are still young. Studies have also discovered that 73 H3.3^G34R^ increases ALT by preventing telomere *KDM4B* histone demethylase, and that *ATRX* mutations are necessary for ALT activation. It has been demonstrated through *in vivo* research that *Kdm4b*^-/-^ and H3.3^G34R^ serve the same purpose in activating the ALT mechanism, and* IDH1*^R132H^ is most likely to act along the same pathway. Additionally, ALT-positive cells with ectopic *KDM4B* expression suffer from telomere degradation. Therefore, the loss of *KDM4B* is required for ALT activation, which can be achieved in the primary cell model by manipulating the four specified variables. Telomerase inactivation, *TP53* checkpoint activation, *ATRX*, and *KDM4B* (H3.3^G34R^ or *Kdm4b*^-/-^) are the four variables that have been identified.

### Pancreatic neuroendocrine tumors (PanNETs)

With a 60% death rate, panNETs are the second most prevalent pancreatic epithelial tumors 132, 133. PanNET is divided into three groups by the WHO: low-grade (G1), intermediate-grade (G2), and high-grade (G3) based on the classification and evaluation of the proliferative rate of tumor cells 133. Although 90% of PanNETs are G1 or G2 tumors, G3 tumors have a higher mortality rate than G1 or G2 tumors 133. PanNETs are mostly sporadic but can possibly be linked to three genetic syndromes: von Hippel-Lindau syndrome, tuberous sclerosis complex, and multiple endocrine neoplasia type 1 (MEN-1) 134.

In this investigation, primary PanNETs that were ALT-positive had larger tumor sizes and a higher pT classification than primary PanNETs that were ALT-negative 135. ALT activation is a late occurrence in PanNET tumors, as evidenced by the observational finding that the prevalence of ALT increased considerably with tumor size 135. Similar outcomes were attained in several experiments, and only PanNET subsets larger than 3 cm showed a decrease of nuclear *ATRX* or *DAXX* expression 128. Only 14.2% of tumors with a diameter of less than 2 cm tested positive for ALT 58. There are notable instances, nevertheless, where nuclear *ATRX* and *DAXX* expression is still present in Alt positive PanNETs 135. These alterations might result from the genetically altered *ATRX* or *DAXX* losing their function, but not from the loss of nuclear protein expression 135. In a similar vein, Wenzel M. Hacking et al. investigation's revealed that the proportion of patients with non-functional PanNETs (NF-PanNETs) rose sharply 136. *ATRX*/*DAXX* loss and the presence of ALT are intimately related to the known poor prognostic characteristics of NF-PanNETs, including large tumors, high WHO grade, lymphatic vascular infiltration, peripheral nerves Invasive, advanced pathological T stage, and regional lymph node metastasis, according to a significant international multi-agency NF-PanNETs cohort study 137. In patients with NF-PanNETs, loss of *ATRX*/*DAXX* and ALT are independent predictive indicators of shorter relapse-free survival 137.

### Leiomyosarcoma

Leiomyosarcoma, which makes up 5% to 10% of all sarcomas 138 is a malignant tumor that differentiates smooth muscle. The 40% survival rate of leiomyosarcoma, an aggressive tumor, is low 139. Myogenic differentiation, which includes smooth and skeletal muscle as well as myofibroblasts, makes sarcomas more aggressive than non-myogenic sarcomas 140, 141.

A highly complex cytogenetic tumor without recurrent chromosomal abnormalities is leiomyosarcoma 139. The survival advantage of chemotherapy for metastatic leiomyosarcoma has not been established, and the majority of patients eventually pass away from the condition 142, 143. The outcomes are particularly bad for deep big tumors and tumors connected to massive blood vessels 144. According to the available data, ALT is a more significant telomere maintenance mechanism in sarcoma than telomerase activation 139. 31 of of 59 leiomyosarcoma cases (or 53%) tested positive for ALT in previous large-scale studies utilizing telomere-specific fish 139. The results of the other two studies were similar; 59% and 62% of the positive samples for leiomyosarcoma were from ALT leiomyosarcoma, respectively 54, 55. Further research has revealed that ALT-positive leiomyosarcoma is linked to tumor necrosis, poor differentiation, epithelioid/polymorphic cell shape, and a high FNCLCC (Federation of the French Cancer Centres) grade 139. One aggressive smooth muscle tumor linked to clinical outcomes is uterine leiomyomas (ULMSs) 145, 146. Several studies that used *in vitro* sequencing to evaluate the genetic mutations of ULMSs. Tumor protein *p53* (6/19; 33%), *ATRX* (5/19; 26%), and mediator complex subunit 12 (*MED12*; 4/19; 21%) are the most frequently altered genes 90. Changes in *TP53* and *MED12*, as opposed to *ATRX* mutations, have frequently been linked to ULMSs 147.

### Malignant fibroushistiocytoma (MFH)

The most prevalent kind of soft tissue sarcoma is MFH. Pleomorphic rhabdomyosarcoma (RM) was the previous name for it 148, 149. It is a relatively uncommon form of cancer that often affects the extremities and infrequently the peritoneum 140, 148. Particularly, MFH growths can occur in the liver or heart on occasion, as well as in the skin, head and neck regions, posterior space, or abdominal cavity 148, 150. MFH is typically detected after it has spread locally or is aggressive 149. Complete resection followed by chemotherapy appears to be a successful course of treatment 150. Although there are currently comparable treatments available, individuals with this condition have a 12-month median survival time 150.

**Figure 4:**
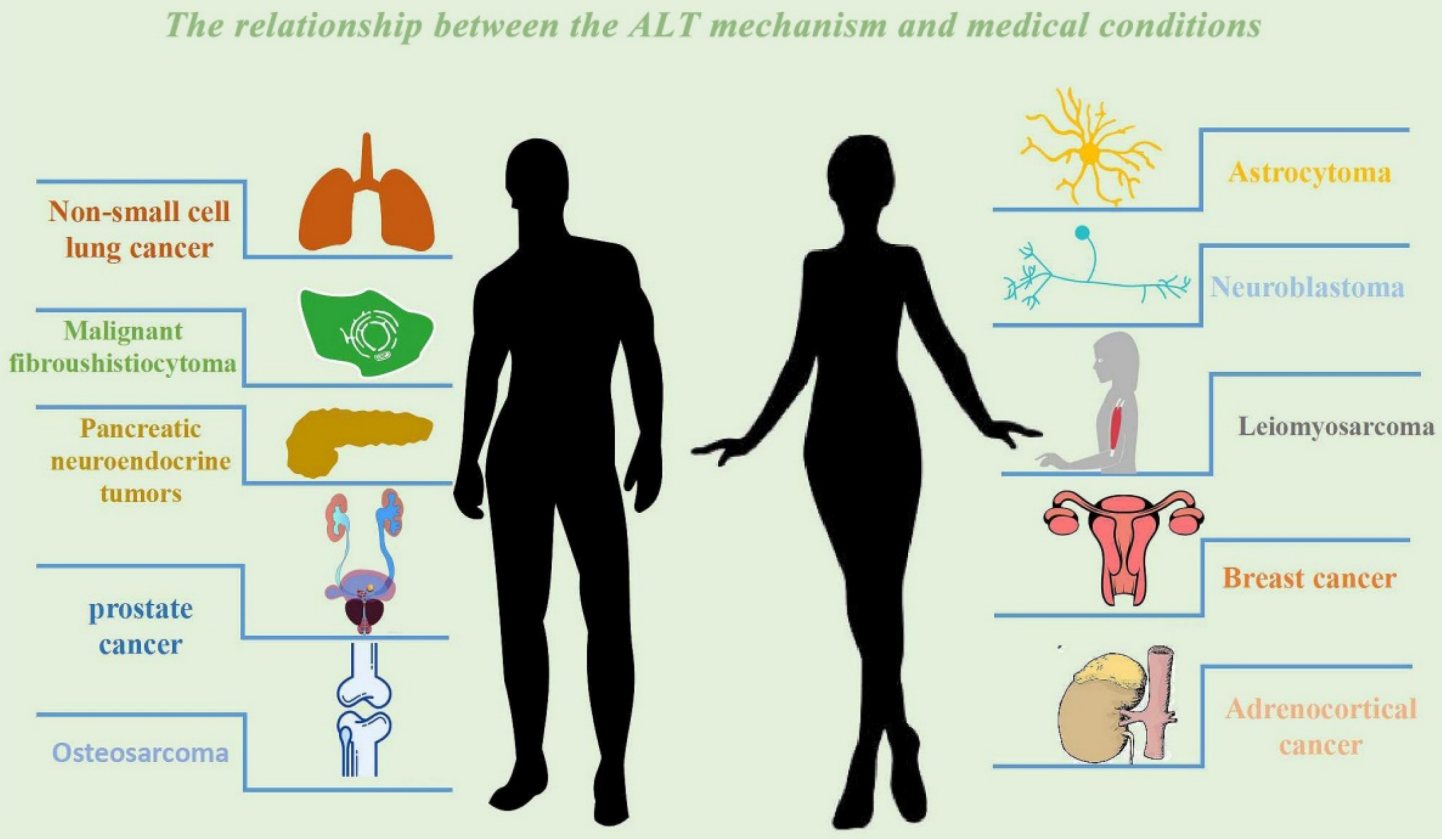
The relationship between the ALT mechanism and medical conditions. The following cancer classes are listed together with the ALT mechanism: Non-small cell lung cancer, Breast cancer, Prostate cancer, Adrenocortical cancer, Neuroblastoma, Osteosarcoma, Astrocytoma. Malignant fibrosarcoma, pancreatic neuroendocrine tumors (PanNETs), and leiomyosarcoma.

Existing data show that 14.3% (32.6%) of the 43 soft tissue malignant fibrous histiocytoma samples tested positive for ALT 151. Complicated karyotypes with particular translocations that exhibit monotonous cell shape as well as complex genetic and chromosomal instability characteristics might be used to categorize sarcoma subtypes 151, 152. Soft tissue tumors with complicated karyotypes are somewhat related with ALT 152. A typical complex karyosarcoma exhibits a low frequency of ALT, demonstrating the varied characteristics of sarcoma, despite research showing that ALT is not the main source of chromosomal instability 54. Although the Meier curve indicates that patients with ALT-positive telomerase-positive tumors have a worse prognosis than those with ALT-negative telomerase-positive tumors (five-year survival rates of 0% and 71.6%, respectively), and that ALT-negative patients have higher average survival rates than ALT-positive patient 151, 153. In the meantime, it was noticed that hTERT expression was found in 90.7% of tumor samples, telomerase activity was found in 79.1% of 43 soft tissue malignant fibrous histiocytoma specimens, and ALT involvement in telomere length maintenance mechanisms was seen in 32.6% of tumor samples 154. The only independent predictive predictor for patient death among the factors examined was the presence of ALT positivity (hazard ratio, 0.275; 95% confidence range, 0.104 to 0.724; p = 0.0089) 154. In a clinical study of a small sample of bone MFH, a rare primary malignancy, telomerase activity and telomere length were measured in 10 MFH specimens using PCR, gel hybridization, and other techniques 155. Similar results were shown. According to the assay analysis, telomerase activity, hTERT expression, and evidence of an ALT mechanism were all found in 100% of the tumor samples, and ALT was a significant predictive risk factor for bone MFH (p=0.0316) 155. The current clinical cohort research on MFH focus more on examining the relationship between the disease's ALT and survival and its course, leaving the investigation of its mechanism in state of obscurity 151, 153. We anticipate that more researchers will pay attention to the MFH and ALT mechanism in the future, investigate its targets, and offer fresh concepts and approaches for the creation of MFH medications **(Fig. 4).**

## ALT tumor inhibitors and possible therapeutic targets in clinical practice

### Telomere shortening of ALT+ cells

### APBs

APBs are PML organisms with ALT ties. The synthesis of APB and ALT telomeres is stopped by the loss of PML, an essential component of APB. APB and ALT telomere synthesis will also be disrupted by BLM helicase loss, whereas BLM overexpression will promote telomere extension and APB expression 156-158. In order to determine if PML and its associated APB are crucial to the ALT pathway and to further investigate the long-term impact of ALT on the preservation of telomere length 159, studies have used the PML null cell line. The findings demonstrated that, despite not being a necessary component for long-term cell viability preservation, PML is necessary for ALT+ cell telomere maintenance 159. In addition, PML is required for the development of the C-cicle, per research findings from PML null cells 159. PML, which includes BLM, is necessary for the positioning of APB components at the ends of telomeres 159. By attracting BTR complexes to the ends of telomeres, PML plays a crucial function in ALT 159.

In human cells, PML-I to PML-VI splicing variations have been identified 160. The ALT+ osteosarcoma cell line U2OS was treated with CRISPR-Cas9 technology to eradicate PML, and a U2OS derivative line devoid of all PML variants was produced 161. This was done in order to establish which PML variants can sustain ALT. Then, in PML KO cells, all six PML variants were expressed 161. All cells that had the PML gene knocked out had significantly reduced telomere production when compared to wild-type cells 161. Only PML-IV among the PML variations may bring back normal levels of telomere synthesis 161. The primary PML variant that functionally promotes ALT is PML-IV, which is the only PML variant that returns telomere clustering to wild-type levels 161.

The topic of whether PML can be a target site for ALT-positive malignancies is therefore deserving of additional research, especially in light of the significant roles that APBs and PML play in telomere extension and the ALT pathway.

### HDR

In ALT cancer cells, telomeres display a distinct nuclear protein structure that results from improper chromatin control, which encourages a cycle of DNA damage and replication stress that activates HDR 162. It is generally known that the non-homologous end joining (NHEJ) or HDR pathway is used in mammalian cells to repair the majority of DSBs 163. Of course, there are some rarely used or alternative mechanisms that also aid in DSB repair, such as single strand annealing (SSA) and alternative NHEJ (alt-NHEJ) 164-166. 80% of DSBs are swiftly repaired by the traditional or classical NHEJ pathway, which predominates in human cells and is active throughout the cell cycle 167, 168. However, due to the requirement for homologous DNA sequences or the availability of sister chromatids as repair templates, HDR is a significantly slower DSB repair process that is restricted to the S and G2 phases of the cell cycle 169. Even though NHEJ makes up a larger portion of DSB repair than HDR does, HDR nonetheless becomes a prominent characteristic of ALT+ cancer cells. The HDR pathway can be summed up as follows 163: (1) the MRN complex binds to each damaged dsDNA end; (2) end excision is carried out by the MRN complex, CtIP, EXO1, BLM, and stabilization of the ssDNA overhang is achieved by binding to RPA; (3) RPA is replaced by RAD51 and Holliday junctions with homologous sequences are formed; and (4) the H End excision generates ssDNA tails, which are then replaced by RPA to create nuclear filaments, which are then replaced by RAD51. The MRN complex (MRE11-RAD50-NBS1), which is necessary for recognizing homologous sequences, is then replaced by RAD51 170. Additionally, BRCA1 and BRCA2 aid in the development of RAD51 nuclear filaments. Once RAD51 has been recruited, a homology search can be carried out 171, 172. If it is successful, the uncut strand can then be allowed to invade the homologous template, allowing the displace template strand to form a displacement-loop(D-loop) 173. Based on the research that have been discussed thus far, we may infer that the HDR pathway plays a significant role in the development of ALT, and that blocking HDR may be a key mechanism to encourage the death of ALT+ cancer cells.

### Inducing ALT+ cell synthesis and lethality

### Ataxia Telangiectasia and Rad3-related (ATR) inhibitor

Recombination plays a role in ALT 14, 174. ALT in cancer is associated with the loss of the chromatin remodeling protein *ATRX* 101, 175. Loss of *ATRX* causes DNA RPA occurs, which generates a recombinant nuclear protein structure and throws off the cell cycle regulation of the telomere non-coding RNA TERRA 176, 177. A crucial stage in DNA replication and homologous recombination (HR) involves single-stranded DNA (ssDNA) covered with RPA 178. During DNA replication, RPA momentarily attaches to telomeres before being released following the S phase. In addition to being an HR intermediate, RPA-ssDNA also attracts ATR nucleoprotein 176, 179. ATR is a crucial protein kinase regulator of an HR, and blocking ATR is essential to preventing RPA recruitment from recombinating 176, 179. Once recruitment and recombination are suppressed, ALT will be greatly reduced, leading to chromosomal division and ALT tumor cells dying 176. As ALT-dependent cancer cells are very selectively killed by ATR inhibitors, this inhibitor may be helpful in the treatment of ALT-positive cancers 176.

### The Fanconi anemia, complementation group M (*FANCM*)

The general view is that a certain level of physiological damage to the DNA is required to sustain the ALT mechanism in order to facilitate telomere extension 180. This indicates that the degree of telomere damage is still below a critical level, where it is sufficient to cause DNA synthesis-based repair but not so great as to cause cell death 180. The ribonuclease RNaseH1 tightly regulates the level of the telomere R-loop, which is created by TERRA and the telomere DNA and can trigger the replication pressure of ALT 41, 181. However, its mechanism is still unclear. According to certain mechanistic studies, the Fanconi Anemia (FA) complex's *FANCM* ATPase/translocase facilitates effective FA complementation group D2 protein (FANCD2) ubiquitination upon replication fork stalling due to physical barriers like DNA crosslinks 182. The biomarker FANCD2, which is thought to be the most precise and quantitative for ALT and encourages BLM recruitment, is necessary for the development of the C-circle. ALT telomerogenesis is induced by replicative stress 3, 183. In addition to altering the replication fork, *FANCM* also suppresses meiotic crossover, attracts DNA repair components to the site of damage, and encourages the activation of ATR checkpoints 180. Depletion of *FANCM* can result in telomere replication pressure because it helps replication forks move through telomere bundles in ALT tumor cells efficiently 180, 184. Additionally, significant replication pressure can also be produced by consuming *FANCM* complex (*FANCM*-FAAP24-MHF1&2) in addition to *FANCM* alone 183. In other words, Fanconi anemia-associated protein 24 kDa (FAAP24) or *FANCM* deficiency causes a marked increase in C-circle formation 180. Insufficient levels of *FANCM* in ALT cells can result in cell death, activation of the ATR signal, high telomere replication stress, and damage 185. In *FANCM* deficient ALT cells, the TERRA R-loop builds up on the telomeres, and downregulating it lowers APBs, replication stress, and C-circle formation 183, 186. Therefore, by limiting the R-loop, *FANCM* enables controlling the activity of ALT and the proliferation of ALT cells 180, 183. Future clinical interventions may target FANCM as a possible target.

### TRF1/TRF2

The production of APB, a distinctive characteristic of ALT cells, depends on sumoylation TRF1 and TRF2, as well as a number of PML-related proteins, such as PML, the MRN complex, RAD52, and RPA 68, 187, 188. The telomere integrity in stem cells and cancer cells may be regulated by a protein called nucleostemini 189, 190. In ALT cells, the reduction of NS enhanced TA and the quantity of telomere damage lesions while decreasing the percentage of telomere damage caused by APB and the quantity of APB 191. DNA damage may increase PML-IV and sulfurized TRF1 recruitment by NS in ALT cells, which is one potential molecular mechanism 190. Previous research has shown that NS knockdown impairs the recruitment of RAD51 and causes spontaneous telomere damage 190. Similar to this, TRF2ΔBΔM (a TRF2 mutant that causes telomere damage by impairing the stability of the telomere complex), telomere length, low telomere signal, and sister chromatid fusion frequency are all increased by NS depletion 157. When NS is overexpressed, TRF2^ΔBΔM^-induced telomere damage in ALT and TA+ cells can be avoided 190. Other research has demonstrated that the SMC family of proteins (SMC1to SMC6) regulate chromosome dynamics by forming three multi-subunit protein complexes 192, 193. The study's findings demonstrate the significance of the Structural Maintenance of Chromosome 5/6 (SMC5/6) Complex in ALT cell telomere maintenance 192, 193. TRF1 and TRF2 are two of the telomere binding proteins that are translated by the SMC5/6 complex SUMO's MMS21 small ubiquitin-like modifier (SUMO) ligase 194, 195. The synthesis of APB requires the sulfidation of many shelterbelt complex subunits, which is stimulated by the MMS21 SUMO ligase 194, 195. Telomere shortening and senescence in ALT cells are caused by the inhibition of telomere HR, which is based on the depletion of SMC5/6 subunits by RNA interference 187. In particular, the SMC5/6 complex promotes the lengthening of telomere HR and ALT cells and the development of APB by vulcanizing telomere binding proteins 187.

### Testis-specific Y-encoded-like protein 5 (TSPYL5)

TSPYL5 is a part of the PML body that co-localizes with ALT telomeres and is essential for the survival of ALT+ cells 196. TSPYL5's ability to combat ubiquitin-specific protease 7 (USP7)-dependent may play a part in its ability to maintain the level of POT1 in ALT+ cells 196, 197. TSPYL5, which is a part of the PML body, can shield POT1 from USP7 and PML-dependent poly-ubiquitination when it is recruited to ALT telomeres 196. When TSPYL5 levels are low, the associated ubiquitin ligase may be activated, leading to the ubiquitination and destruction of POT1 via the proteasome 198. The N- and C-terminal regions of USP7 may directly interact with PML proteins I and IV to explain this mechanism 199. Without TSPYL5, the interaction of PML and USP7 may aid in the recruitment of PML and E3 ligases to the telomeres and the degradation of POT1 200. These ligases include RING finger protein 4 (RNF4), ubiquitin-like with PHD and RING finger domains 1 (UHRF1), mouse double minute 2 (MDM2), and tripartite motif-containing 27 (TRIM27) 200. Early research has suggested that USP7-dependent poly-ubiquitination may be related to the loss of *ATRX* function in ALT+ cells, and that alterations in POT1 brought on by degradation interact with *DAXX* 196. In summary, preventing the connection between TSPYL5 and USP7 may present novel therapeutic chances to specifically cause cell death in ALT+ malignancies with little adverse effects on healthy normal tissues 196.

### BTR

**Figure 5:**
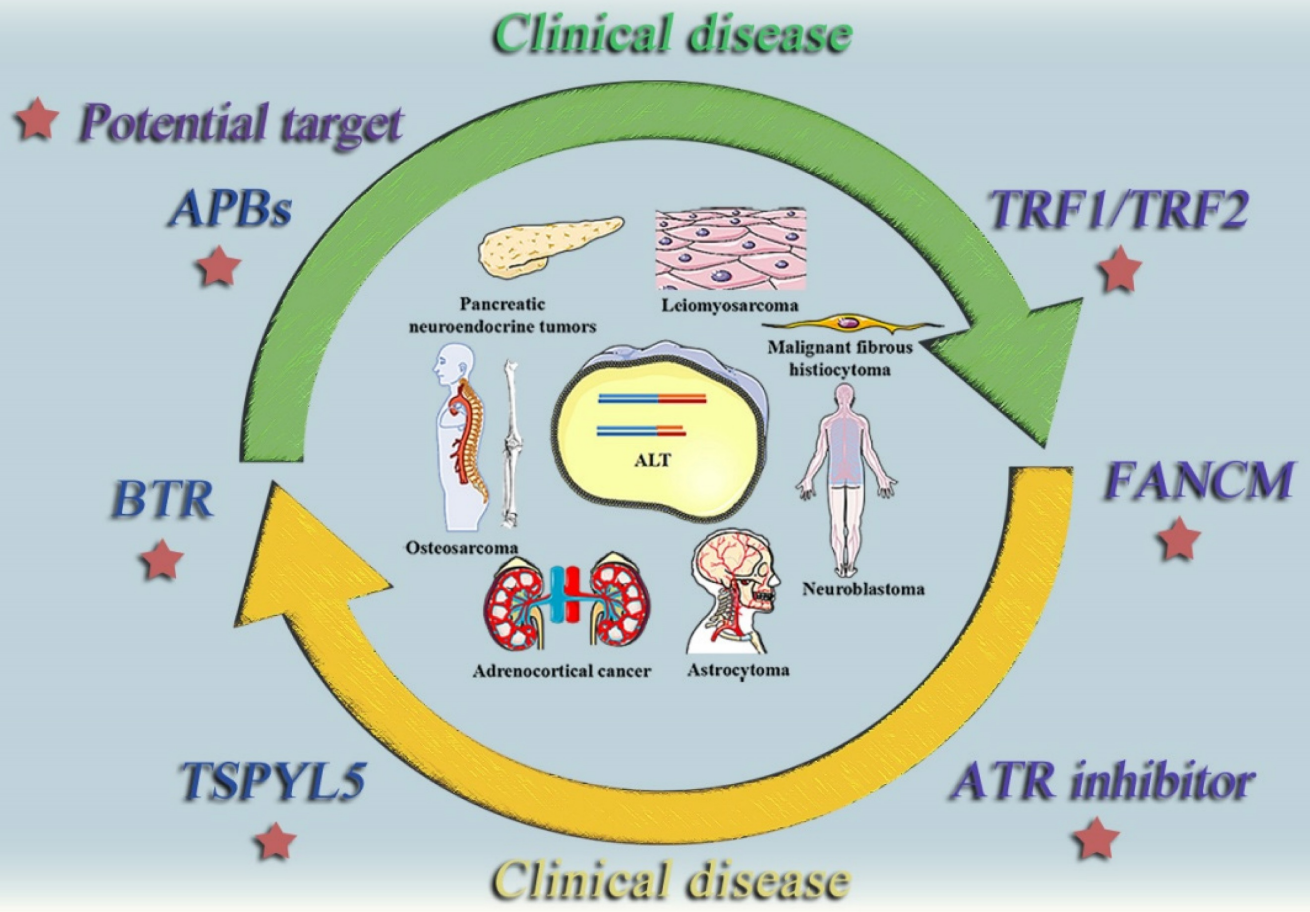
ALT positive tumor diseases and potential therapeutic targets. This diagram summarizes some typically clinical tumor diseases that are currently believed to be regulated by ALT mechanism and potential therapeutic targets that may be effective in the future.

The presence of PML nucleosomes that are tailored for the APB is one of the characteristics of ALT cells 159. APB collects with telomeres and DNA damage factors at the ends of telomeres. According to several research, PML is necessary for the ALT mechanism 159. This is required because APB is involved in directing the BTR complex to the end of ALT telomeres 159. Surprisingly, the BTR complex is recruited to telomeres without the assistance of PML, demonstrating that BTR localization to telomeres is sufficient to maintain ALT activity 159. In fact, cells lose important ALT signals such telomere length heterogeneity, extrachromosomal C-circle formation, and telomere synthesis in G2/M when PML is absent, which eventually causes telomeres to gradually shorten 161, 201. The study's findings indicate that the process of APB production in ALT cells encourages the buildup of BTR, which in turn encourages the break-induced replication-mediated telomere elongation 159. The SUMOization of PML is widely regarded as being essential to its function in ALT 202, 203. Telomeres are moved to the nuclear pore by SUMOylation in order to encourage telomere elongation 202, 203. Nuclear polySUMO peptides elicit ALT-like properties in a BLM-dependent way when telomeres are artificially aggregated. The BTR complex at the telomeres is sufficient to induce telomere ALT, increasing the likelihood of BTR recruitment, and it is sufficient to maintain ALT activity without the need for APB components or epigenetic changes 159. A quick and targeted death of ALT cells can be brought on by the erosion of the BTR complex 159 **(Fig. 5).**

## Conclusion

Telomeres are well known to be crucial in controlling cell development, aging, and death. The growth of tumor cells is also influenced by the length of telomeres in tumor cells. Telomerase lengthens telomeres, one of the two currently acknowledged methods of telomere lengthening; the other is the ALT mechanism. Naturally, ALT tumor cells exhibit certain common traits. These biomarkers aid in determining if the ALT process occurs and, in some cases, maintain its stability. According to earlier research, activation of the ALT pathway has been noted in various tumor disorders. These tumor patients frequently have complex diseases, refractory illnesses, and low overall survival times. Some genes or proteins have been identified as playing a crucial part in the molecular mechanism of ALT tumor cells, and as a result, they may one day serve as viable therapeutic targets for ALT tumor cells.

The definition of ALT, typical ALT tumor cell traits, putative molecular causes of ALT, ALT-related tumor disorders, and potential clinical treatment targets were the main topics of this review. This review aims to deliver partial theoretical information for future ALT investigations by combining the findings of outstanding preliminary studies.

## Figures and Tables

**Table 1:** ALT incidence in various cancer type

Number	Cancer types	ALT+ rate	References
No.1	Osteosarcoma	64%	53, 54
No.2	Synovial sarcoma	9%	53, 54
No.3	Malignant fibrous histiocytoma	62%	53-56
No.4	Leiomyosarcoma	58%	53-56
No.5	Liposarcoma	25%	53-56
No.6	PanNET	53%	53, 55, 57, 58
No.7	Paraganglioma	13%	53, 55, 57, 58
No.8	Carcinoid tumor	6%	53, 55, 57, 58
No.9	Neuroblastoma	34%	8, 53, 55
No.10	Ganglioneuroblastoma	14%	8, 53, 55
No.11	Adrenocortical carcinoma	12%	8, 53, 55
No.12	Astrocytoma	42%	53, 55, 59
No.13	Glioblastoma	28%	53, 55, 59
No.14	MSI-H Gastric Carcinoma	57%	53, 60
No.15	Non-MSI-H Gastric Carcinoma	19%	53, 60
No.16	Intestinal cancers	6%	57
